# Don’t be reviewer 2! Reflections on writing effective peer review comments

**DOI:** 10.1007/s40037-021-00670-z

**Published:** 2021-06-11

**Authors:** Chris Watling, Shiphra Ginsburg, Lorelei Lingard

**Affiliations:** 1 Department of Oncology and Centre for Education Research and Innovation, Schulich School of Medicine and Dentistry, Western University, London, ON Canada; 2 Department of Medicine, Sinai Health System and Temerty Faculty of Medicine, University of Toronto, Wilson Centre for Research in Education, University of Toronto, Toronto, ON Canada; 3 Department of Medicine and Centre for Education Research and Innovation, Schulich School of Medicine and Dentistry, Western University, London, ON Canada

In the Writer’s Craft section, we offer simple tips to improve your writing in one of three areas: Energy, Clarity and Persuasiveness. Each entry focuses on a key writing feature or strategy, illustrates how it commonly goes wrong, teaches the grammatical underpinnings necessary to understand it and offers suggestions to wield it effectively. We encourage readers to share comments on or suggestions for this section on Twitter, using the hashtag: #how’syourwriting?

We recently had a manuscript rejected from a journal following peer review. That in itself is an occurrence neither unusual nor tragic. We all experience rejection, and often the peer review process improves our work so that we can resubmit and contribute more convincingly to the scholarly conversation. But this time the experience felt different. This time it felt *personal*. We were left feeling deflated, belittled, and irritated—not exactly the optimal frame of mind for retooling a rejected paper.

Peer review can sting. It is intended as a collegial, respectful enterprise, but the popular “Reviewer 2” meme in social media suggests that it often feels otherwise [1, 2]. Reviewer 2 symbolizes the peer reviewer who is rude, vague, smug, committed to pet issues, theories, and methodologies, and unwilling to treat the authors as peers. A recent linguistic analysis of such reviews found features such as attitude markers (e.g., verbs like “reject”, sentence adverbs like “absurdly”, and adjectives like “illogical”), self-mention (e.g., “I cannot possibly imagine”), and boosters (e.g., “the manuscript is utterly ridiculous”) [3].

Reviews exemplifying such characteristics are hard to take and can have a number of negative consequences for the authors, the journal, and the field. Reviewer 2 can push the imposter syndrome button for both novice and more experienced writers, causing them to doubt their scientific abilities. They can consign papers with good potential to a desk drawer for eternity. They can cost journals future submissions and the field future knowledge. And perhaps most disturbingly, they can disproportionately harm underrepresented groups. A 2019 study found that intersectional groups such as women of colour and non-binary people of colour were most likely to report direct negative impacts on scientific aptitude, productivity, and career advancement after receiving an unprofessional peer review [4]. In an effort to help us all avoid being Reviewer 2, this Writer’s Craft reflects on what makes constructive peer review comments so tricky to write and offers suggestions to make your next peer review clearer, more collegial, and more efficient.

## Recognizing the challenge

Peer review is voluntary, unpaid, and often unrecognized and unrewarded work by busy academics and clinicians. We’re squeezing this work in on evenings and weekends, and perhaps feeling resentful as we do about the time stolen from our own writing projects [5]. But peer review is also a critical community service, and one with multiple aims: it serves the field, the journal, and the authors. In the service of the field, peer review intends to uphold shared standards to ensure that our combined knowledge progresses robustly. In the service of the journal, peer review assesses the paper’s potential to contribute originally to an ongoing conversation. In the service of the authors, peer review supports them to achieve both—the high standards of the field and the meaningful advancement of a journal’s scholarly conversation. Writing peer review comments with three aims in mind, however, is complicated, particularly when you are recommending major revision or rejection. And since outright acceptance is vanishingly rare, feedback to the authors about how to improve is almost always tangled up with assessment to the editor about how to proceed regarding acceptance. So the first step as a reviewer is to recognize the challenge of multiple aims and audiences, and to write each section of your review with a clear sense of which of these aims and audiences you’re prioritizing, and why.

## Making it meaningful

The author and the journal’s editor need different things from your review, and so the risk that someone will be disappointed—or disheartened—is high. The author hopes for feedback that will strengthen their scholarship, while the journal wants an assessment of the paper’s quality and suitability for publication. How can we reconcile the sometimes-competing purposes of our reviews to ensure they are meaningful? Let’s consider some helpful writing strategies.

### Your conversation with the author

Treat the feedback-forward parts of the review as a conversation, writing as though you were sitting down with the author(s) to talk about their work. The second person, used rarely in academic writing, works well here to build rapport and create a sense of intimacy. For example, notice how the third person approach creates distance and evokes a sense of a harsh judgment:

*The authors do not sufficiently explain themselves in the Methods section, which is jargon-filled*.

Perhaps you felt defensive just reading that line! In contrast, an approach that uses first- (“I”) and second-person (“you”) pronouns feels more like a conversation with a colleague:

*I stumbled over some of the jargon in your Methods section; I’d suggest that you adopt more plain-language explanations to reduce the risk of losing your reader*.

Remember that effective feedback should be specific and actionable [6]. Don’t leave the author in suspense. If an Introduction is suffering the absence of one or two key citations, name them if you can, rather than settling for a vague dismissal like *“There is a vast literature on this topic that the authors have ignored.”* If the Discussion fails to revisit one of the Introduction’s core concepts, call that out explicitly. Instead of *“The Discussion is underwhelming and fails to show how the study has moved the needle in this domain”*, try something more directive, such as *“I’d suggest returning more clearly in the Discussion to the issue of patient-centred care and how this study challenges some of its core principles, given the prominence of that line of argument in the Introduction.”*

Meaningful feedback also targets the task and not the person [7]. Steer clear of personal swipes at the researchers, even if you think their paper falls short. Perhaps you are tempted to write something like this:

*The authors seem oblivious to the extensive existing literature on this subject in the field of higher education, and thus claim their discoveries as original when they are not*.

Take a breath—and consider revising in a way that redirects the feedback away from the “oblivious” authors and toward a piece of work that could be strengthened. While the version below remains unmistakably critical, its critique is directed at the paper and not at the author(s):

*The paper’s claim to originality is weakened by its lack of reference to similar work done in the field of higher education*.

You’ll also notice that this comment deliberately deviates from the use of the second person to achieve this redirect.

Such redirection is a form of hedging, which refers to a toolbox of linguistic and rhetorical moves that allow us to express caution, uncertainty, and politeness. Hedging is necessary and abundant in scientific writing [8, 9], and it also has a place in peer review. Hedging acts to save face [10], buffering the author whose work is being reviewed against threats to their self-esteem, and protecting the reviewer from being perceived as arrogant or dismissive. These impacts matter, because without them feedback is harder to accept and use. Hedging positions authors to be open to your suggestions. It doesn’t imply evading hard truths or avoiding critique. Rather, it recognizes that when feedback is baldly face-threatening or tramples on self-esteem, it is less likely to be effective in shaping real improvements in the work.

### Your conversation with the editor

While it’s helpful to think of the review primarily as a conversation with the author, remember that the journal’s editors (chief and/or associate) will be listening in. Every line of your review need not be construed as feedback for the author. From time to time, you need to ensure that the editor will hear your concerns clearly, understand how much weight you assign to those concerns, and appreciate the rationale for your ultimate recommendation. Most journals ask reviewers to start with a few comments reflecting their overall impression of the paper, and these comments are as much for the editor as for the authors. Here’s your chance to say something like:

*The authors have done a rigorous piece of work that addresses a pressing question in health professions education. While I have identified a number of opportunities for the paper to be strengthened, I find it a compelling piece of work that represents a novel contribution to the literature on this issue, and I look forward to seeing it in print*.

This opening salvo lets the associate editor know that you think the paper is strong and that the journal would do well to publish it. With that context in mind, the associate editor need not try to read between the lines of your recommendation. They will understand—even if you suggest a significant revision—that any subsequent critiques are not fatal flaws, but rather opportunities for improvement. Many journals also include a confidential comments box that reviewers can use to flag up issues to the editor. Reviewers may use this box in a variety of situations, such as when they’re worried about ethical or methodological issues, uncertain about the decision they’ve recommended, or compelled to contextualize their review comments. Be careful, though, that you’re not contradicting your reviews in this space, or using it for critiques that your bland, uncritical reviews avoid mentioning. Editors are in a tough spot when the reviewer comments don’t support a final decision that was prompted by confidential comments.

The first few sentences of your review of each section of the paper might similarly contain messages for the associate editor that reflect your judgment and the intensity of your feeling. For example:

*While this Introduction is well written, I struggle to identify the gap in the literature that this study seeks to fill. The literature review is comprehensive but rather uncritical, creating the impression that we already know everything there is to know about this problem. This issue contributes to my main concern about this paper: I’m just not persuaded that the work is sufficiently original to merit publication*.

Here, the issues raised are clearly weighty and threaten the paper’s acceptability for publication. The associate editor will no doubt get the message. At the same time, hedging strategies soften the blow for the authors. The concerns are directed at the product (“the Introduction”, “this paper”) rather than at its producers. Positives (“well written” and “comprehensive”) balance the critiques, and modifiers allow for some uncertainty in the judgments that are offered (“*rather* uncritical”, “not *sufficiently* original”). And the use of “I struggle” and “I’m not persuaded” reminds us that a review is, after all, opinion and not universal truth.

## Think big picture

While it may be tempting to point out every single sentence or idea that you disagree with, don’t. Authors may feel like you are ‘piling on’ the critique, and it will be difficult for them to sift through to find what is essential. Instead, hit the high points and don’t nitpick. The high points include whether or not the researchers have tackled a problem that matters, have used appropriate and well-conducted methods, have written a story that is coherent, and have situated their work in the existing conversation. You are not a copy editor. Particularly when the decision is to reject, don’t drown the authors in all the typos, comma splices, and improperly formatted references that you noticed. If the language overall is difficult to navigate as a reader, make a summary comment about that with a few examples. Something like “*I notice that the authors use single and double quotation marks inconsistently in the manuscript, which can be distracting for readers. I’d suggest a careful proofread with this issue in mind before resubmitting*” may help the authors more than a detailed list of every instance of sloppy punctuation.

Reviews can consume several hours of the reviewer’s time. We think this should be reconsidered, as it may contribute to the resentment and reviewer burnout we highlighted above. One strategy is to take an hour to read the paper and make some notes, and then set it aside. Taking a pause like this is especially important if the paper has evoked an emotional response, which can lead to some of the nastier or more personal comments described above. Then take, at most, another hour to craft your review. In our combined experience, if it seems to be taking much longer than this, it may be a sign that the paper has too many flaws and will ultimately be rejected. If this is the case, it may be more helpful for you (and for the authors) to streamline your comments and focus on hitting the high points. If your reviews regularly take more than a couple of hours, it is possible that you are overstepping your role. You are not the authors’ supervisor or thesis advisor, and while it may feel instinctive to take an educational approach, it may not actually be helpful for the authors or editors.

Another strategy to help the authors and editors interpret your comments is to carefully distinguish between your reflections and your requests. Reflections are nice, as they show the authors that you are deeply engaging with their work. But keep these reflections brief. We have seen reviews that run longer than the paper they are commenting on, which in turn require similarly lengthy responses back from the authors: this can put both authors and editors in a difficult position, especially if word limits are an issue. In the end, what matters are your suggestions for revision: actionable requests for more, less, or different material in certain parts of the paper. It may be helpful to demarcate these two sections clearly, so the authors know specifically what to respond to.

Finally, we offer a few quick tips to help you avoid some of the pitfalls of peer review commentary.

### Don’t police

You may wish the authors had conducted another study. They didn’t. So don’t request it. Certain peccadillos may bother you, but don’t fixate on them or allow yourself to view them as fatal flaws. Consider what can be fixed with clearer writing and what cannot—for example, poor study design or low response rates cannot be corrected at this stage. If these flaws are dealbreakers, point that out honestly to the authors and the editor. But there are relatively few fatal flaws, and things like an incomplete description of a coding process or insufficient explanation of a theory rarely count as such. Remember that the editors and readers will also cast a critical eye on the work; you needn’t see yourself as the last word on the study.

### Beware your ego

As our discussion of hedging suggested, feedback is more effective if it isn’t personal. That goes for the feedback writer as well as the feedback recipient. Frustrated that your work didn’t make it onto the author’s citation list? Let it go. Self-citation requests abound in peer review and may be a questionable practice. One study reported that 44% of requests for additional citations were self-citations [11]. If the omission is truly a gaping hole in the literature review, make it clear why and provide the citation. Authors shouldn’t have to guess which of your papers you think is relevant. Surprised to find a completely different orientation to a problem you’ve studied? Or taken aback that your work is explicitly challenged by the paper? Set aside the instinct to protect your turf and engage with the ideas on their own terms. If you cannot, perhaps you should recuse yourself as a reviewer.

### Identify yourself

Reviews can be blinded, meaning the reviewers do not know who the authors are, and anonymous, meaning authors don’t know who their reviewers are. Identifying yourself puts you in the rhetorical position of talking to the writer, which ought to trigger the same diplomacy skills you’d use when standing up to ask a question following a conference presentation. (If you lack such skills altogether, you probably have no business engaging in peer review.) In an effort to improve the quality and tone of reviews, some journals now offer “open peer review” in which reviewers can opt (or are required, depending on the journal) to sign their reviews. There are power issues to be considered here: reviewers earlier in their career may fear the consequences of unblinded reviewer comments, particularly for work written by senior members of the community.

### Learn from others

Some journals now share with all reviewers both the editor’s decision and the full set of reviews. Not only does this make reviewers more publicly accountable for their remarks, but it also affords them the opportunity to read other reviewers’ comments. We have found this approach quite educational. We get to see when other reviewers respond differently than we did, which reminds us that interpretation is key. We note that other reviews have perceived different problems and strengths than the ones our review focused on. And sometimes we pick up turns of phrase that are particularly effective, and we unashamedly steal them for future application when we need to make difficult peer review feedback more digestible.

## In summary

Peer review can be thankless work. But imagine that it wasn’t. Imagine that you are writing a review that the author will genuinely thank you for. Maybe not today … but someday. If the paper is accepted, you want to feel that you’ve played a small role in helping to strengthen it, and the author should feel a bit prouder of their work. And even if the paper is rejected, you’d like the authors to feel they have gained something of value. For some authors, the idea that a reviewer really engaged with and took their writing seriously is affirming, even if the decision is to reject. So don’t be a Reviewer 2 who leaves writers disillusioned and discouraged. Be that reviewer who engages and encourages. That reviewer who, a year hence, might get invited for coffee at a conference as gratitude for the role you played in strengthening the author’s manuscript.
